# Conditions Which Promote or Retard the Progress of Dental Caries

**Published:** 1889-06

**Authors:** C. N. Peirce

**Affiliations:** Philadelphia


### CONDITIONS WHICH PROMOTE OR RETARD THE PROGRESS OF DENTAL CARIES.*

BY C. N. PEIRCE, D.D.S., PHILADELPHIA.

The pathological condition designated " Dental Caries " is certainly one of the most frequent and most serious affections to which the teeth are liable, and educated dentists are all united in characterizing it as a " progressive and often continuous softening from the exterior to the interior of the crown, until a larger portion, or the whole of the tooth affected has gradually disappeared." Much of the labor of the dentist is employed in more or less successful efforts to modify, or arrest the progress of this destructive disease, and to restore as far as possible the ravages it has made in the organs under his care.

To do this intelligently, and with the most favorable results, many things are to be comprehended, and their influence appreciated and wisely heeded. If we attempt to tear down or disintegrate any structure, organic or inorganic, this labor is facilitated by a knowledge of the tissues or molecules of which it is composed, and of the manner of construction; so, also, if the attempt be made to stay or modify the influence of the antagonisms of its environment, the same knowledge is helpful to the success of such efforts.

---

* Read before the Odontological Society of Pennsylvania, March, 2d, 1889.

In pursuance of this thought, first to be recognized, are the facts that in the segmentation and aggregation of living cells, primitive integuments are formed, and from one of these the tooth germs have their origin, and that the processes through which this germination takes place are always liable to modification by the *interruption of nutritive distribution.* Histologists note also, that the impulse which establishes the individuality of the tissues, as well as that of the organ, must be given to it while in this immature and plastic condition, and that now also must the laws controlling heredity and adaptation to function, exert their morphological or formative influence, and through these and the nutritive currents, must be evolved the differentiated structures resulting in the ameloblasts, odontoblasts, and other tissues which are so essential to the development of the complex tooth.

After months of vital activity and continuous change, what were simple homogeneous cellular structures, become complex heterogeneous tissues, and efforts at specialization have resulted in a dissimilarity preparatory to a most remarkable process of solidification. In this latter process, the previously mentioned ameloblasts and odontoblasts (specialized cells only), in their functional duties have produced structures marvellous in density and (with aided vision) in beauty; and when these are normal in shape, and continuity, they are unequaled in their power of mechanical resistance by any other organic structures.

The morphology of the individual enamel cell, fiber or prism, is not arbitrary, or the result of a whim or accident, but a mechanical necessity, essential to the density of the structure. What other shape but hexagonal could they be to make a compact and solid structure of normal shape and size? Who would not recognize the spaces in a bundle of cylindrical bodies, however tightly they may be bound together? And, if brought under pressure sufficient for solidification, the hexagonal shape must most nearly be approximated in the individual integrals. The crown of dentine, upon which these enamel cells rest, with the line of their axis almost vertical to the coronal surface, is not a plane in any sense of the term, but is made up of wavy or irregular elevations and depressions, and upon this uneven foundation these cells must rest, varying in the line of their perpendicular position, and in their density, the latter quality being in correspondence with their near or remote position regarding nutritive supply, and *their previous, as well as their prospective, functional activity.* The normality of this enamel tissue, with its maximum resisting power, is dependent largely upon the general or systemic condition during the period of its development and calcification. Its vices of conformation and pathological predispositions, which make this tissue an easy prey to an acid environment, are exhibited in a want of continuity of the cells, and an imperfect calcification of the same, inducing pits and ragged or roughened depressions, all which are factors in dental caries, through their power of absorbing, and retaining in contact with the tissue, a destructible agent; hence it is of the utmost importance that the influences of these possible conditions should be recognized and appreciated.

The dentine structure, possessing a much larger per cent. of organic matter, is necessarily, by virtue of this, less dense than the enamel; and though originating like the enamel from a homogeneous cellular germ, it is evolved only through a multitude of progressive changes, culminating in a double layer of highly specialized odontoblastic cells, and their conspicuous prolongations, covering the coronal surface of the plastic mass, known as the dentine matrix or papilla. These odontoblastic cells are recognized to be the final effort of the plastic cellular mass, previous to the depositions, or secretions, of the salts of lime; and to be themselves the active agents in the elimination of the mineral substance which forms the tissue known as dentine.

The dentinal portion of a tooth is of the three dental tissues the most constant, and yet the most varied in its histological development, displaying structural peculiarities in the same species, and marked differences in different species. It affords the solid foundation upon which the enamel fibers rest, and conveys to them about all the nutrition and sensation they possess, after the tooth crown has once been thoroughly deprived of its vascular covering by complete eruption. Indeed, it is the only medium through which the enamel can have any arterial or nervous connection, after it has passed through its enveloping sack and the gum, except the little it may receive through the thin margin of cementum, with which it comes in contact on the neck of the tooth. The tubuli, which permeate the dentine, and open with their largest diameters upon the pulp chamber, giving an impetus to the ingress of fluids, are, when the tooth is in normal condition, a source of strength and nutrition; but when the organ is attacked with caries, they become a source of weakness, and facilitate its decalcification and the subsequent decomposition of the organic matter which has served as a matrix for the inorganic. The dentine, like the enamel, is liable to imperfections, or structural defects, from disturbed or mal-nutrition, a sequence of constitutional abnormalities. These defects may be slight or serious, varying with the severity or duration of the disturbance.

They may consist of masses of semi-calcified dentine, or of cells or spaces known as "interglobular spaces," distributed throughout the body of dentine; though they are more frequently recognized near the zonal line, between the enamel and dentine, which makes them more important factors in favoring decay. The infolding upon themselves of the terminal ends of the tubuli, and the more frequent diseases of childhood, are probably two of the causes to which this appearance or condition may be attributed.

These recognized vices in structure are entirely harmless, while the mail, which nature has provided for the dentine, remains intact; but when the enamel rods become broken, decalcified, or otherwise disturbed, so as to admit the ingress of a solvent, they become actively accessory to dentine decalcification, and subsequent disintegration, as do also the tubules themselves; and they do this through their power, first of admitting the solvent, and second of affording habitat and protection to organisms which consume and disintegrate the organic material of the tooth, and supply as their product a mass which re-acts as a solvent upon the salts in the surrounding structure.

The cementum which covers the dentine of the root and is itself covered and nourished by the cemental or periodontal membrane, is less dense than the dentine, possessing a larger percentage of organic matter than does that tissue. It is, as compared with enamel and dentine seldom attacked by dental caries, for the reason, that when in normal condition, it is protected by its membranous covering. But when by virtue of location and surrounding influences, it becomes the seat of caries, the increased amount of organic matter it possesses and the pabulum and protection it offers to minute beings, greatly favors its progress and that too with more than usual discomfort to the patient. In connection with, or under the gingival borders of the gums and cervical margins of the alveolar process, it is not only an accessible cavity to fluids, but one that encourages the lodgment or impaction of food which in its fermentation, facilitates the advancement of the disease. In its structural arrangement cementum differs from dentine, but not in a manner to protect it from the ingress of fluids, when once its lacunae and canaliculi are exposed.

The organic matter of these two tissues, dentine and cementum, by continuity and interlacing of their fibers with one an other, is a source of sensation or nervous impulse and nutrition to the former dentine. This union and sympathy so frequently manifested' between dentine and cementum is by dentists recognized in a tooth in which the pulp has been devitalized.

The chemical composition and predispositions of these dense dental tissues, must not be overlooked in a paper on dental caries. The fact that tooth density is almost wholly due to the presence of the salts of lime, and also that some of the acids which are at times found in the mouth, either as a result of systemic or of local conditions, readily act upon this inorganic material is important, and must be noted as a factor concerning the possible chemical changes taking place in the oral cavity. The secretions of the mouth, which, without cessation, are being emptied into it from its numerous glands, are of a constantly varying quantity and quality being subject to these modifications, through both systemic and local condition.

But it matters not what the origin of their normality or their abnomality, of their benign or their vicious influence, the effect upon the teeth is the same, and dental physiologists, and pathologists, must recognize in the saliva, an important factor in favoring or in modifying the progress of dental caries.

To ascertain with any degree of certainty the influence the fluid is at the time exerting upon the teeth, access can and should be had to some convenient and simple test, such as litmus paper, which when turned from blue to red, or restored to blue from red, gives with a good degree of certainty the acidity or alkalinity of the secretion.

It has just been stated that this salivary secretion varies through either systemic or local conditions.

The first inquiry that naturally arises from this statement is whether there is any one period in the individual's life, so far as age is concerned, when there is a greater tendency to an acid abnormality than another.

Second, whether there is any abnormal systemic condition that may favor or induce this unfavorable condition of the oral fluids.

Third, whether there are any physiological processes which may induce the same destructive tendencies in their secretion.

That the observing dentist recognizes a greater tendency to dental caries in the young than in mature or middle life, needs only to be suggested to be appreciated, and that this is partially or indeed largely due to a prevaling acidity of the saliva is quite as patent. But the *reason* for the prevailing abnormality of this fluid is not quite so clear—youth or immaturity *per se*, would not be a satisfactory explanation, so we must look for conditions accompanying this.

It is not probable that in this period of growth the demand made upon the nutritive currents for the building of the osseous srtuctures would exhaust their supply of calciferous element, and hence so rob the blood of this ingredient that the secretions would on the slightest provocation present an acid reaction, and, again, with this minimum quantity of the essential salt, would not fermentations also be more active and their results manifest greater vigor, thereby producing that condition, so almost universally observed in the youth of both sexes, viz., rapidly progressing dental caries?

The lines of systemic normality and abnormality run so nearly parallel and contiguous, that the slightest deviation of the former may encroach upon and assume the rôle of the latter with all its attendant consequences. It is of little moment so far as the influence it may exert upon the oral secretions is concerned, whether this deviation be caused by states of fatigue—mental or physical—by anaemia from deficiency in quantity or quality of blood, by malaria, or by still more serious deviations, complicated with typhoid conditions. These abnormalities, would, one and all, if of sufficient duration, record their presence upon tooth structure, to be modified only by present or previous local conditions which would have power to retain their activity and destructive influences.

Physiological processes, may by taxing localities or organs, induce an irritability of the nervous system, or they may instigate what in medical parlance is termed nervous prostration, which acts very unhappily, not only upon tooth environment, but also upon the resisting power of the tooth itself; so that during periods of gestation and lactation as well as periods of prolonged intense mental application and anxiety, the dentist's labors are not only in greater demand, but his skill is taxed to its utmost, to keep such patients comfortable until the much needed operations can be better performed and sustained.

The effect of the above enumerated conditions is frequently first recognized through the accumulations upon the teeth. If these are of an acid and soft cheesy consistency, it is safe to assert that the dental tissues are suffering or soon will suffer from their environment, unless some remedy be applied to counteract its influence. On the other hand, a change from these abnormal systemic conditions to a more healthy physical state, is soon predicted by a change in th e nature and consistency of the deposits upon the teeth. If these should be firm in attachment, dense in structure, and varying in color from a dark cream to a black, dental caries, if progressing at all, is not from the influence of the glandular secretions, but from a product of the fermenting accumulations of food or debris, in the interstices or sulci in the crowns, or spaces between the same ; in a word, from want of cleanliness. These conditions and their results are so readily observed, that with safety the assertion can be made, that the presence or absence of tartar or salivary calculus, or its tendency to accumulate, or the reverse, is of great significance regarding the progress of dental caries.

In recognizing the variety of influences that may promote or retard the progress of dental caries, the fact must not be overlooked that they do not all act simultaneously, nor are they all necessarily of equal duration. Conditions which may be termed predisposing causes of decay may be present in a marked degree in the form of vices of conformation, deficiency in quantity and quality of enamel and dentine, yet in as conspicuous a degree may decalcification of these abnormally developed tissues be absent. And again, the exciting cause of caries may be present in the nature of abnormal secretions, and their influence may be stayed or greatly modified by a previous condition of tooth structure, by cleanliness, and ant-acids and anti-septics. The vitality of the tooth, and its recuperative power, are factors here which must not be overlooked, for the resistance which they offer in their antagonism to decay is important.

The principles involved in prophylactic treatment, and the proper selection of agents exerting such influence, should not be disregarded. The value of therapeutic measures when systemically indicated must be recognized.

In a word, the educated, intelligent and successful practitioner of dentistry needs to know, not only that teeth do decay, but the nature and source of the solvent and its antidotes.

He needs to know, not only that certain vices of conformation predispose to decay, but the causes of such malformations, the possibility of anticipating them, and the best method of protecting them when they exist.

He needs to know, not only that certain systemic conditions vitiate the secretions, but through what functional derangement they are engendered, and whether they can be corrected or anticipated and their influence modified.

He needs to know, not only that the tooth has a recuperative power, but at what age it is active, how it protects the tooth, and what systemic conditions favor and what annul or abort its influence.

These things he needs to know so that he may work intelligently, with pleasure to himself, with profit to his patients, and with the most economical expenditure of nervous energy for both patient and operator.

